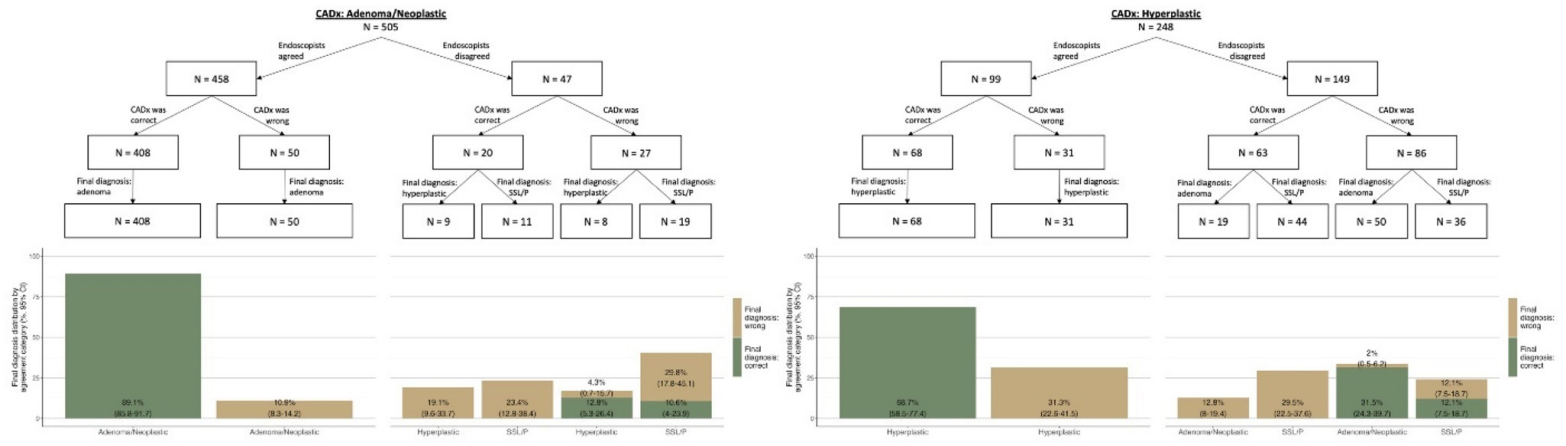# Poster Session I - A54 HUMAN-MACHINE INTERACTION IN OPTICAL POLYP DIAGNOSIS: DECISION-MAKING AFTER CADX POLYP DIAGNOSIS

**DOI:** 10.1093/jcag/gwaf042.054

**Published:** 2026-02-13

**Authors:** D Dubois, Y Jalal, H Pohl, D K Rex, C Hassan, R Djinbachian, M Oleksiw, V Michal, D von Renteln

**Affiliations:** Gastroenterology, Centre Hospitalier de l’Universite de Montreal, Montreal, QC, Canada; Gastroenterology, Centre Hospitalier de l’Universite de Montreal, Montreal, QC, Canada; White River Junction VA Medical Center, White River Junction, VT; Indiana University School of Medicine, Indianapolis, IN; IRCCS Humanitas Research Hospital, Rozzano, Lombardia, Italy; Gastroenterology, Centre Hospitalier de l’Universite de Montreal, Montreal, QC, Canada; Centre Hospitalier de l’Universite de Montreal, Montreal, QC, Canada; Gastroenterology, Centre Hospitalier de l’Universite de Montreal, Montreal, QC, Canada; Gastroenterology, Centre Hospitalier de l’Universite de Montreal, Montreal, QC, Canada

## Abstract

**Background:**

Computer-assisted diagnosis (CADx) systems employ artificial intelligence algorithms to generate probabilistic outputs, assisting in polyp characterization. The clinical utility of these AI-generated predictions ultimately depends on the endoscopist’s ability to interpret and appropriately act upon them. CADx demands expertise from endoscopists, who must employ knowledge of polyp histology based on surface features and clinical judgement to accept or reject the AI-based polyp classifications. This requires a higher level of endoscopist knowledge to discern whether an AI-generated classification is accurate and should be incorporated into the final diagnosis or whether it requires correction.

**Aims:**

Our study aimed to evaluate endoscopists’ decision-making when interpreting CADx predictions for each polyp category, compared to histopathology diagnosis as ground truth. Specifically, we examined the accuracy of accepted and rejected CADx outputs, the frequency and direction of overrides, and the patterns of reclassification following rejection.

**Methods:**

We conducted a secondary analysis of a prospective cohort between 2022-2025 (NCT06822816) in which CADx was used during colonoscopies to classify polyps in real-time as either neoplastic or hyperplastic. Endoscopists could either accept the diagnosis made by CADx or reject it by providing an alternate prediction. They could chose to predict polyp histology as adenoma, hyperplastic or sessile serrated lesion. This histology prediction was made prior to resection and independent of pathology. All small (<10mm) polyps with documented CADx output, endoscopist histology prediction, and adenoma, hyperplastic or SSL pathology diagnosis were included.

**Results:**

Our cohort included 754 polyps from 367 patients. Accepted CADx diagnoses were correct for 89.1% of neoplastic and 68.7% of hyperplastic CADx predictions. Endoscopists accepted incorrect classifications more frequently for hyperplastic than neoplastic polyps (31.3% vs.10.9%). Upon rejecting correct CADx neoplastic predictions, endoscopists misclassified 23.4% as sessile serrated lesions. Similarly, 29.5% of rejected correct CADx hyperplastic predictions were incorrectly reassigned as SSLs.

**Conclusions:**

Endoscopists demonstrate a pronounced diagnostic bias towards neoplastic lesions, frequently misclassifying hyperplastic polyps as SSLs. While clinically safe, this phenomenon might have implications increasing downstream endoscopy procedures and cost-effectiveness of CADx, highlighting the necessity of incorporating comprehensive cost analyses including downstream colonoscopy utilization. Developing CADx systems with robust SSL identification capabilities will likely be critical in overcoming these diagnostic challenges and achieving effective integration into routine clinical practice.

**Funding Agencies:**

None